# Statistical reanalysis of vascular event outcomes in primary and secondary vascular prevention trials

**DOI:** 10.1186/s12874-021-01388-6

**Published:** 2021-10-17

**Authors:** Lisa J. Woodhouse, Alan A. Montgomery, Jonathan Mant, Barry R. Davis, Ale Algra, Jean-Louis Mas, Jan A. Staessen, Lutgarde Thijs, Andrew Tonkin, Adrienne Kirby, Stuart J. Pocock, John Chalmers, Graeme J. Hankey, J. David Spence, Peter Sandercock, Hans-Christoph Diener, Shinichiro Uchiyama, Nikola Sprigg, Philip M. Bath

**Affiliations:** 1 Stroke, Mental Health & Clinical Neurosciences, University of Nottingham, Nottingham, UK; 2 Nottingham Clinical Trials Unit, University of Nottingham, Nottingham, UK; 3 Department of Public Health and Primary Care, University of Cambridge, Cambridge, UK; 4 The University of Texas Health Science Center at Houston, Houston, USA; 5 University Medical Center Utrecht, Utrecht, Netherlands; 6 Hopital Sainte-Anne, Université Paris-Descartes, Paris, France; 7 Department of Cardiovascular Sciences, Universitaire Ziekenhuizen Leuven, Leuven, Belgium; 8 Chronic Disease & Aging Unit, Monash University, Clayton, Australia; 9 NHMRC Clinical Trials Centre, University of Sydney, Sydney, Australia; 10 London School of Hygiene & Tropical Medicine, London, UK; 11 George Institute for Global Health, Sydney, Australia; 12 Department of Neurology, University of Western Australia, Crawley, Australia; 13 Robarts Research Institute, London, Canada; 14 Centre for Clinical Brain Sciences, University of Edinburgh, Edinburgh, UK; 15 Department of Neurology, University of Duisburg-Essen, Duisburg, Germany; 16 International University of Health and Welfare, Otawara, Tochigi Japan

**Keywords:** Prevention, Vascular event, Analysis, Ordinal

## Abstract

**Background:**

Vascular prevention trials typically use dichotomous event outcomes although this may be inefficient statistically and gives no indication of event severity. We assessed whether ordinal outcomes would be more efficient and how to best analyse them.

**Methods:**

Chief investigators of vascular prevention randomised controlled trials that showed evidence of either benefit or harm, or were included in a systematic review that overall showed benefit or harm, shared individual participant data from their trials. Ordered categorical versions of vascular event outcomes (such as stroke and myocardial infarction) were analysed using 15 statistical techniques and their results then ranked, with the result with the smallest *p*-value given the smallest rank. Friedman and Duncan’s multiple range tests were performed to assess differences between tests by comparing the average ranks for each statistical test.

**Results:**

Data from 35 trials (254,223 participants) were shared with the collaboration. 13 trials had more than two treatment arms, resulting in 59 comparisons. Analysis approaches (Mann Whitney U, ordinal logistic regression, multiple regression, bootstrapping) that used ordinal outcome data had a smaller average rank and therefore appeared to be more efficient statistically than those that analysed the original binary outcomes.

**Conclusions:**

Ordinal vascular outcome measures appear to be more efficient statistically than binary outcomes and provide information on the severity of event. We suggest a potential role for using ordinal outcomes in vascular prevention trials.

**Supplementary Information:**

The online version contains supplementary material available at 10.1186/s12874-021-01388-6.

## Introduction

Effective vascular event prevention lies in the management of modifiable risk factors, and also treating the causes of an initial event in the case of secondary prevention. There are numerous prevention strategies to reduce the risk of cardiovascular outcome events such as stroke, myocardial infarction (MI) and bleeding. These strategies include reducing blood pressure [1–3] and cholesterol [4, 5], and the use of antiplatelets [6, 7], anticoagulation [8], surgery [9, 10], and vitamins [11, 12] to improve outcome. Some interventions may be hazardous and increase vascular risk, for example hormone replacement therapy (HRT) [13, 14].

Effective primary and secondary prevention results in a lower absolute risk of vascular events. As absolute event rates are a key component in calculating sample sizes for binary event outcomes, lower event rates mean larger, longer and more expensive clinical trials [15]. Further, there has been an increase in the numbers of clinical trials being undertaken, due to new therapies being tested. This combination of more and larger clinical trials means recruitment of the required number of participants is a difficult and competitive process [16]. Therefore, new strategies are needed to reduce clinical trial sample size, which will reduce costs, time to completion and number of participants exposed to risks.

One possible approach is to analyse vascular prevention trials in a way which incorporates more data that could also be considered as clinically relevant. Most vascular prevention trials compare binary event rates between the treatment and control group. However, vascular event outcomes such as stroke, myocardial infarction (MI) and bleeding, can be fatal or nonfatal, this generating trichotomous outcomes (a variable with three levels; i.e. fatal event / non-fatal event / no event). Further, non-fatal events may have different severities, so that further extensions to the ordinal outcome may be included to generate four or more levels of outcomes [17, 18]. Analysis of this type of ordered categorical event is likely to be more efficient statistically than that of dichotomous outcomes. This opens up the potential for reducing trial sample size or detecting smaller but still clinically-relevant benefits. Such structuring of vascular event outcomes assumes that the ordering of events is meaningful, i.e. fatal vascular events are considered more severe than nonfatal events. Ordered categorical outcomes could also be more informative to participants and healthcare professionals than binary ones, [17] e.g. rather than saying that an intervention reduces the risk of stroke, we can say that it reduces both stroke and the severity of stroke events.

We have previously performed an empirical analysis of published summary data taken from the primary publications of 101 vascular prevention trials that supported the above concepts [17]. Here, we report a prospective study based on analysis of individual participant data. Specifically, we compared the relative statistical efficiencies of ordinal versus binary outcomes as part of the Optimising the Analysis of vascular Prevention trials Collaboration. Although the use of statistical approaches for ordinal data is well defined in the methodological literature, its use for designing and analysing vascular prevention trials is novel. We also report on the effect of adjusting for baseline characteristics on the efficiency of the analysis methods.

## Methods

### Identification of trials

This study followed the methodology used for the optimising the analysis of acute stroke trials collaboration [19, 20]. The protocol for this research has been published elsewhere [21]. We sought individual participant data from randomised controlled trials assessing the primary and secondary prevention of vascular events. Potentially eligible studies were identified electronically through search engines including the Cochrane Library and PubMed (to the end of 2016). Further information on the search strategy is given in Supplementary Table 1. The use of relevant filters within these search engines was also utilised to help narrow the search for randomised controlled trials. Once trials were identified, one author (LJW), reviewed the abstracts (and results sections if necessary) to determine if the trial was eligible. Trials were included if they showed benefit or harm according to the trial publication, or were included in a meta-analysis showing benefit or harm. Trials were excluded if they were neutral (showed no significant effect on the primary outcome) and were part of a neutral meta-analysis. Trials were also excluded if they only collected data relating to the occurrence of events and not any information regarding the severity of the event.

### Data sharing

For each eligible study, lead researchers were contacted by an email that included the study protocol and invited them to join the collaboration and share their data. Up to 4 reminders were sent if the researcher did not respond. In some cases, data were obtained via application to data repositories maintained either by the trial funding body (e.g. National Institute of Neurological Disorders and Stroke; National Heart, Lung and Blood Institute) or hosting commercial trial data (clinicalstudydatarequest.com).

### Trial data

Shared data included information on participant demographics (age, sex, medical history), trial design (setting, intervention, length of follow-up) and vascular events/outcomes (stroke, MI, bleeding), including information on the severity of those events (fatal, non-fatal, severe, mild). In trials where there were more than two treatment groups (e.g. factorial trials), outcomes were analysed for each treatment comparison that had been performed in the trial’s main publication. Data were analysed according to allocated treatment using observed outcome data only and no imputation was performed.

### Formation of outcomes

Ordered categorical outcomes were created for each available event (stroke, MI, major adverse cardiovascular events, bleeding; Supplementary Table 4). For example, stroke was categorised into 3, 4, 5, 6, 8 and 9-levels defined by severity.

### Statistical analysis

After a review of published vascular prevention trials regarding methods for analysing ordinalised outcomes, fifteen different statistical methods were chosen for analysing treatment effects. Methods included binary logistic regression (adjusted), Cox Proportional hazards (adjusted and unadjusted) and Chi-Square test for the binary outcome measures, and 2xN Chi-square test, Cochran-Armitage trend test, ordinal logistic regression (adjusted and unadjusted), Mann-Whitney U test, Median test, t-test, multiple linear regression (adjusted) and bootstrapping the mean rank for the ordinal outcomes [22–26]. The Win-ratio test, which is a method where multiple binary outcomes, with varying levels of clinical importance, can be analysed together to determine a ‘Win ratio’ (calculated as wins/losses), was also used for this study [27]. An overview of the chosen methods can be seen in Table 1. For regression-based analyses, both unadjusted and adjusted analyses were performed; adjustments were made for variables common to all data sets: age, sex and history of diabetes. No transformations of outcome were performed for any of the analysis methods. Analyses were carried out in SAS (version 9.3).

**Table 1:** Review of statistical analysis methods [22–27]

Analysis method	Outcome type	Statistical assumptions	Advantages	Disadvantages
Binary logistic regression (BLR)	Binary	• No assumptions made about explanatory variables	• Can adjust for covariates	• Large number of observations required
Cox proportional hazards (CPH)	Binary	• Proportionality of hazards over time • Censoring of observations is unrelated to prognosis	• Can adjust for covariates	• If assumptions of the model not met then subsequent analyses and risk estimates will possibly be biased
Chi-square (χ^2^) (CS)	Binary and ordered categorical	• Chi-Square – Total count is > 40 or total count is 20–40 and the expected value of each exposure-outcome category is > 5	• Simple to implement	• Cannot adjust for covariates
Cochran-Armitage trend test (CAT)	Ordered categorical	• Similar to the Chi-square test but it takes into account the ordering across categories	• Easy to interpret	• Cannot adjust for covariates
Ordinal logistic regression (OLR)	Ordered categorical	• Response is ordinal • Proportionality of odds	• Can adjust for covariates	• If assumptions of the model not met then subsequent analyses and odds estimates will possibly be biased
Mann-Whitney U test (MWU)	Ordered categorical	• Non-parametric test • Response is ordinal / continuous • Observations from both groups are independent of one another	• Easy to interpret	• Cannot adjust for covariates – there are extensions of this method, which allow for adjustment [28–30]
Median test (MT)	Ordered categorical	• Non-parametric test • Considers the position of each observation relative to the overall median.	• Easy to interpret	• Cannot adjust for covariates • Inefficient (low power) to detect differences if sample size is large.
t-test	Continuous (used on the ordered categorical)	• Response is continuous • Homogeneity of variances	• Easy to interpret	• Cannot adjust for covariates
Multiple linear regression (MLR)	Continuous (used on the ordered categorical)	• Response is continuous • Linear relationship • Homogeneity of variances • No or little multicollinearity	• Can adjust for covariates	• Assumes linear relationship • Sensitive to outliers
Win Ratio testWins/losses version (WR)	Combination of binary outcomes	• Responses for each outcome are binary • Accounts for clinical priorities of endpoints	• Prioritises the more major component of the outcome • Useful for composite outcomes • Extensions of this approach allow for covariate adjustment [31] • Easy to interpret	• New method • Doesn’t use the precise times from randomisation to event occurrence
Bootstrapping (BS)	Ordered categorical	• None	• No assumptions made about the distribution of the data	• Cannot adjust for covariates • Computationally intensive • Doesn’t provide a meaningful point estimate

### Comparison of statistical tests

Each trial comparator dataset was analysed using each statistical approach; an example of the application of this methodology can be seen in Supplementary Table 5. The results of the tests for the ordinal outcome and the binary counterpart were then ordered within each dataset and given a rank (numbers 1 to 15), with the smallest rank given to the test that produced the smallest probability value (i.e. 2-sided *p*-value, with the exception of the 2xN Chi-Square) within that dataset. A 2-way analysis of variance test, ANOVA-Friedman with adjustment for ties [32], was used to determine if there was a difference between the average ranking of each test across the datasets. If the ANOVA test was significant, Duncan’s multiple range test [33] was then performed to assess the ordering of tests and to assess where significant differences between tests were present. The results of the Duncan’s multiple range test for the 3-level extensions of the MI and bleeding outcomes were then more closely inspected. As for the stroke outcomes, the stroke/TIA 4-level outcome was chosen for closer inspection, rather than the 3-level, as it contained more severity information and the fact that there were more comparator datasets available for it than for any of the other stroke outcomes. Furthermore, for this outcome boxplots were created to show the distributions of the *p*-value rankings and the *p*-values themselves, respectively, across all of the available comparator datasets, for each analysis technique used.

### Assessments of validity and reliability

A number of supplementary analyses were performed to assess the validity and reliability of the results. Firstly, the comparison of the statistical approaches was repeated within subgroups of trials that shared similar characteristics to assess whether certain methods were more efficient for certain types of trials. Secondly, sample sizes generated using the formulas for the ordinal/continuous statistical tests were compared to those generated using a formula for binary proportions test to determine the effect of outcome/method choice on sample size. Thirdly, the statistical assumptions of the tests were assessed to determine if the use of these tests in these circumstances was appropriate.

The sensitivity, or type I error, of the two most efficient statistical tests (i.e. with the smallest mean rank) were also assessed using ordinal vascular outcomes from 10 randomly selected comparator datasets. For each of the selected comparator datasets we created a dummy treatment variable with a neutral treatment effect so that any treatment difference could only occur by chance. We then, from each of the comparator datasets, generated 1000 sample datasets, using random sampling with replacement. Tests maintaining an acceptable proportion of type I errors would expect to see a significant result in around 50 of the 1000 sample data sets.

## Results

### Trial characteristics

**Fig. 1:**
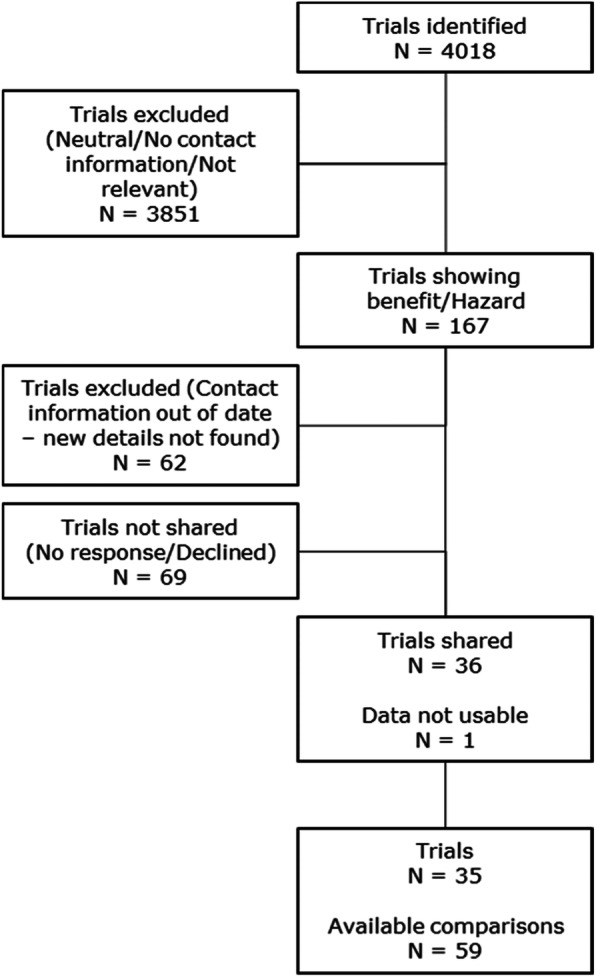
Flow diagram – Identification of included trials

Of 167 identified trials (Fig. 1), data were shared with the collaboration for 35 trials; including 254,223 participants, Supplementary Tables 2 and 3. Reasons for not obtaining data included inability to contact the chief investigator or other investigators (e.g. emails bounced); chief investigators did not respond to requests for data sharing despite multiple attempts and when emails had not bounced; chief investigators explicitly chose not to share their data; and trial data were not available in a usable format, a problem particularly for some older trials. Of the included trials, 15 were primary prevention and 20 secondary prevention (Table 2). Interventions included anticoagulants (ACT; 8 trials), antihypertensives (AHT; 8 trials), antiplatelets (APT; 7 trials), carotid stenting/endarterectomy (CEA; 3 trials), glucose lowering (GL; 1 trial), hormone replacement therapy (HRT; 2 trials), statins (4 trials) and vitamins (2 trials). From the 35 trials, a total of 59 comparator datasets were derived with 13 trials having more than two comparator arms (Fig. 1). Further information regarding the included trials is given in Supplementary Tables 2 and 3.

**Table 2:** Characteristics of trial participants by type of intervention

	Trials	All	ACT	AHT	APT	CEA	GL	HRT	Statins	Vitamins	P
**All**			*N* = 8	N = 8	*N* = 7	*N* = 3	*N* = 1	*N* = 2	*N* = 4	N = 2	
Primary (%)	15		4 (36.7%)	6 (40.0%)	0	1 (6.7%)	1 (6.7%)	2 (13.3%)	1 (6.7%)	0	
Secondary (%)	20		4 (20.0%)	2 (10.0%)	7 (35.0%)	2 (10.0%)	0	0	3 (15.0%)	2 (10.0%)	
**Baseline**											
Number	35	254,223	54,713	81,058	39,714	5074	10,251	27,347	24,222	11,844	
Age	35	65.1 (30.5)	69.0 (11.7)	64.6 (51.9)	66.0 (9.6)	66.6 (8.2)	62.8 (6.6)	63.4 (7.2)	59.3 (8.1)	63.7 (12.1)	< 0.0001
Sex, Female (%)	35	117,222 (46.1%)	21,063 (38.5%)	39,213 (48.4%)	14,697 (37.0%)	2711 (53.4%)	3952 (38.6%)	27,347 (100%)	3916 (16.2%)	4323 (36.5%)	< 0.0001†
Medical History (%)											
Diabetes	34	51,131 (20.1%)	7030 (12.9%)	17,143 (21.2%)	8897 (22.4%)	1135 (22.4%)	10,251 (100%)	1980 (7.2%)	1725 (7.1%)	2970 (25.3%)	< 0.0001‡
Hypertension	32	153,427 (69.6%)	22,771 (67.0%)	76,550 (94.5%)	25,871 (65.2%)	3212 (63.3%)	–	8433 (34.0%)	8141 (33.6%)	8449 (72.0%)	< 0.0001
Hyperlipidaemia	17	27,627 (29.8%)	96 (9.6%)	1019 (15.4%)	14,757 (37.3%)	1291 (37.8%)	–	3366 (13.7%)	4444 (40.2%)	2654 (41.7%)	< 0.0001
IHD	12	27,850 (30.6%)	–	8813 (24.0%)	6724 (20.7%)	1970 (38.8%)	–	–	9014 (100%)	1329 (17.0%)	< 0.0001¥
Stroke	28	21,645 (12.6%)	3403 (9.6%)	5375 (13.9%)	8707 (23.9%)	717 (21.0%)	630 (6.1%)	306 (1.1%)	369 (4.1%)	2138 (18.2%)	< 0.0001
MI	25	22,069 (13.6%)	5073 (14.4%)	704 (2.1%)	2720 (7.7%)	640 (18.8%)	1590 (15.5%)	634 (2.3%)	10,708 (60.8%)	–	< 0.0001
Smoking, Current	31	48,441 (20.9%)	7030 (21.0%)	17,515 (21.9%)	10,211 (25.8%)	1471 (29.0%)	1247 (12.2%)	2831 (10.5%)	3496 (14.4%)	4640 (39.5%)	< 0.0001
Alcohol	12	27,897 (41.3%)	6373 (34.2%)	4754 (48.0%)	10,984 (40.5%)	561 (33.8%)	–	–	3098 (46.9%)	2127 (59.4%)	< 0.0001
SBP	31	145.8 (23.1)	143.5 (27.1)	155.2 (19.8)	147.4 (20.2)	146.1 (19.3)	–	128.7 (17.6)	136.5 (18.8)	141.5 (20.9)	< 0.0001
DBP	30	83.3 (12.0)	79.5 (12.8)	87.7 (11.1)	84.9 (11.7)	80.1 (10.0)	–	76.0 (9.2)	80.3 (10.4)	81.6 (12.1)	< 0.0001
HR	14	73.1 (13.7)	75.1 (15.8)	77.5 (11.6)	73.2 (12.4)	73.1 (10.6)	–	–	67.8 (11.1)	–	< 0.0001
**Secondary**											
Qualifying event (%)											
Stroke	19	71,178 (66.4%)	19,779 (60.9%)	5632 (85.2%)	34,071 (85.8%)	1571 (46.0%)	–	–	–	10,125 (86.1%)	< 0.0001
MI	19	25,528 (22.9%)	12,090 (37.2%)	–	–	–	–	–	1348 (76.3%)	–	< 0.0001
TIA	19	9979 (9.3%)	347 (1.1%)	974 (14.7%)	5497 (13.8%)	1759 (51.6%)	–	–	–	1402 (11.9%)	< 0.0001
OTR	18	91.2 (246.2)	3.2 (12.9)	418.0 (439.6)	31.0 (163.5)	47.9 (43.6)	–	–	507.4 (317.0)	60.2 (178.0)	< 0.0001

ACT: anticoagulants; AHT: antihypertensives; APT: antiplatelets; CEA: carotid endarterectomy; DBP: diastolic blood pressure; GL: glucose lowering; HRT: hormone replacement therapy; IHD: ischaemic heart disease; MI: myocardial infarction; HR: heart rate; OTR: onset to randomisation; SBP: systolic blood pressure; TIA: transient ischaemic attack. Percentages (%) are out of the total number of participants with available data. Comparisons by Chi-Square test and ANOVA. †Excluding HRT group. ‡Excluding GL group. ¥Excluding Statins group

### Participant characteristics

Baseline characteristics of the 254,223 participants are provided in Table 2. For secondary prevention trials, one had information on qualifying event missing, and two did not have time to randomisation data. The most consistently collected baseline data included information on age, sex and history of diabetes. Baseline characteristics differed between intervention types, including age (statin trials, mean 59 years, anticoagulation 69 years); female sex (statin 16%, HRT 100%); history of diabetes (statin 7%, glucose lowering 100%); history of hypertension (statins 34%, antihypertensive 95%); and time to randomisation (for secondary prevention trials, anticoagulation 3 days, statins 507 days).

### Stroke

The derived categorical stroke outcomes ranged between 3, 4, 5 and 8-levels; addition of TIA increased this to 4, 5, 6 and 9 levels (Supplementary Table 4). Comparison of analyses for all levels of stroke outcome found that ordinal analyses were rated higher than binary for 4 and more levels (Table 3). For the 3-level stroke outcome review, ordinal and binary analysis methods appeared to have similar efficiency with adjusted binary logistic regression being rated the most efficient analysis method. Further, adjusted analyses were more efficient with lower ranks then their univariable counterparts, e.g. for OLR, CPH (Table 3). The results of performing the Duncan’s multiple range test on the Stroke/TIA 4-level outcome can be seen in Table 4. Ordinal analyses were rated superior to binary, with the most efficient being the Mann-Whitney U test and ordinal logistic regression (both unadjusted and adjusted); Table 4, Supplementary Fig. 1a. These findings are also supported by the distribution of the *p*-values from each of the tests across each of the datasets, which can be seen in Supplementary Fig. 1b.

**Table 3:** Rating of statistical tests

Outcome	Levels^†^	Comparator datasets	P	Rating of tests relative to each other														
				MWU	Adj. OLR	WR	OLR	BS	Adj. MLR	Adj. BLR	Adj. CPH	TT	CAT	CPH	MT	CSB	CSO	CSF
Stroke	3	56	< 0.0001	**2**	**4**	**3**	7	9	10	**1**	**5**	13	12	**6**	11	8	14	15
	4	23	< 0.0001	**2**	**3**	**1**	**6**	10	12	**4**	**5**	11	13	**7**	8	9	14	15
	5	16	0.0002	**2**	**3**	**1**	**6**	**8**	12	**4**	**5**	11	13	**7**	10	9	14	15
	8	12	0.0115	**3**	**2**	**1**	**7**	**8**	10	**4**	**5**	13	14	**6**	9	11	12	15
Stroke/TIA	4	35	< 0.0001	**1**	**3**	**5**	**2**	**4**	8	10	12	7	**6**	11	9	13	14	15
	5	17	< 0.0001	**1**	**3**	9	**7**	**2**	**4**	10	11	**6**	**5**	12	**8**	13	14	15
	6	13	< 0.0001	**1**	**2**	9	**3**	**6**	**4**	10	11	**7**	**8**	12	**5**	13	14	15
	9	12	< 0.0001	**1**	**2**	7	**3**	**4**	**6**	11	10	8	9	12	**5**	14	13	15
MI	3	47	0.010	**1**	**6**	12	**5**	**2**	**7**	**8**	11	**4**	**3**	10	14	13	**9**	15
Bleeding	3	32	< 0.0001	**4**	**9**	**3**	11	**7**	**1**	**8**	**6**	**5**	**2**	**10**	15	14	14	13
	4	26	0.035	**3**	**7**	**5**	**8**	**2**	**1**	**10**	**9**	**6**	**4**	11	13	14	12	15
	5	13	0.032	**6**	**8**	**5**	**3**	**2**	**1**	**11**	**7**	**4**	**10**	**12**	15	14	**9**	13
Vascular	3	47	< 0.0001	**2**	**3**	**5**	**6**	10	8	**1**	**4**	11	12	**7**	13	9	14	15

The numbers in bold represent the tests that are the most efficient and do not differ statistically from one another. The P-value is from the results of the Friedman ANOVA. The order of the rating of test is based on the mean rank calculated by the Duncan’s multiple range test; the most efficient test (i.e. the test with the smallest mean rank) is rated the best with a score of 1 and the least efficient with a score of 15

Abbreviations

Adj.: adjusted; BLR: binary logistic regression; BS: bootstrapping; CAT: Cochran-Armitage trend test; CPH: Cox proportional hazards; CSB: Chi-square binary event outcome; CSF: Chi-square binary fatal event outcome; CSO: Chi-square ordinal event outcome; MLR: multiple linear regression; MT: median test; MWU: Mann-Whitney U test; OLR: ordinal logistic regression; TT: t-test; Vascular: combination of stroke and MI; WR: win ratio test

^†^Defined in Supplementary Table 4.

**Tables 4:** Results from the Duncan’s test analysis of p-value ranks for 4-level stroke/TIA based on 35 comparator datasets (p < 0.0001)

Test								Mean rank
MWU (4-level)	A							4.69
OLR (4-level)	A	B						6.26
Adj. OLR (4-level)	A	B	C					6.31
Bootstrapping (4-level)	A	B	C					6.43
Win Ratio*	A	B	C					6.69
CA Trend (4-level)	A	B	C					6.71
t-test (4-level)		B	C					6.89
Adj. MLR (4-level)		B	C					6.91
Median test (4-level)		B	C	D				8.06
Adj. BLR (Binary)			C	D	E			8.49
CPH (Binary)				D	E			9.06
Adj. CPH (Binary)				D	E	F		9.34
Chi-square (Binary)					E	F		10.37
Chi-square (4-level)						F	G	11.14
Chi-square (Binary fatal)							G	12.66

Abbreviations

BLR: binary logistic regression; CA trend: Cochran-Armitage trend test; CPH: Cox proportional hazards model; MLR: multiple linear regression; MWU: Mann-Whitney U test; OLR: ordinal logistic regression

*Combined binary outcomes including (from most to least clinically important): fatal stroke (Yes/No), Non-fatal stroke (Yes/No), and transient ischaemic attack (Yes/No).

### Myocardial infarction

Data on 3-level MI (fatal / non-fatal / none) were available for 33 trials (47 comparator datasets). Comparison of analysis methods suggested that there was a significant difference between the tests (*p* = 0.010) and that ordinal/continuous approaches were rated more efficient than binary methods, with the exception of adjusted binary logistic regression (Table 3, Supplementary Table 6).

### Bleeding

Bleeding data were available for 15 (43%) trials and it was possible to create 3-level, 4-level and 5-level outcomes (32, 26, and 13 comparator datasets respectively); Supplementary Table 4. For each of these three bleeding outcomes, ordinal/continuous analyses were more efficient than binary methods. Adjusted multiple linear regression, was the top-rated analysis in each case (Table 3, Supplementary Table 7).

### Major adverse vascular event (composite vascular event)

A composite outcome (fatal stroke or MI / non-fatal stroke or MI / none) was derived for 33 trials; 47 comparator datasets. The comparison of analyses (Table 3) suggested that there was a significant difference between the tests (*p* < 0.0001). The most efficient method appeared to be adjusted binary logistic regression, however this approach did not differ significantly from the Mann-Whitney U test and adjusted and unadjusted ordinal logistic regression suggesting that ordinal approaches were just as efficient as binary methods.

### Subgroup analysis

The results of the comparisons using the 4-level stroke/TIA outcome, for subgroups including type of trial, and the main intervention can be seen in Table 5 and Supplementary Fig. 2; insufficient data were present for glucose lowering and vitamin based trials. The differences between the tests were significant for each of the trial type subgroups (both p < 0.0001) and the top performing analyses appeared to be Mann-Whitney U test, the win ratio test, adjusted ordinal logistic regression and the Cochran-Armitage trend test. Analysis methods also differed for interventions, including ACT, AHT, APT, CEA and statins, with the best rated being the Mann-Whitney U test, bootstrapping, the win ratio test and adjusted ordinal logistic regression.

**Table 5:** Rating of tests by subgroups for stroke/TIA 4-level (35 comparator datasets)

Subgroup	Comparator datasets	P-value	Rating of tests relative to each other														
			MWU	Adj. OLR	WR	OLR	BS	Adj. MLR	Adj. BLR	Adj. CPH	TT	CAT	CPH	MT	CSB	CSO	CSF
*Trial type*																	
Primary	15	< 0.0001	**1**	**6**	**3**	**7**	**8**	**4**	9	12	**5**	**2**	11	10	13	15	14
Secondary	20	< 0.0001	**1**	**4**	5	**3**	**2**	9	10	12	6	8	11	7	14	13	15
*Main Intervention*																	
ACT	10	0.0089	**1**	**9**	**3**	**7**	**10**	**5**	**6**	**11**	**4**	**2**	**8**	14	13	12	15
AHT	6	0.011	**3**	**9**	**7**	**8**	**1**	**4**	**6**	**10**	**2**	**5**	**11**	13	**12**	15	14
APT	10	< 0.0001	**1**	**2**	9	**3**	**4**	**7**	12	11	**6**	**8**	10	**5**	14	13	15
CEA	2	< 0.0001	**5**	**3**	**6**	**4**	**1**	7	11	14	8	9	13	**2**	12	10	15
HRT	2	0.35	4	2	1	5	10	3	12	6	7	8	11	9	13	15	14
Statins	4	0.0011	**1**	**3**	**4**	**2**	**6**	9	12	8	11	7	10	**5**	13	14	15

The numbers in bold represent the tests are the most efficient and do not differ statistically from one another. The P-value is from the results of the Friedman ANOVA. The order of the rating of test is based on the mean rank calculated by the Duncan’s multiple range test; the most efficient test (i.e. the test with the smallest mean rank) is rated the best with a score of 1 and the least efficient with a score of 15

Abbreviations

ACT: anticoagulants; Adj.: adjusted; AHT: antihypertensives; APT: antiplatelets; BLR: binary logistic regression; BS: bootstrapping; CAT: Cochran-Armitage Trend test; CEA: carotid Endarterectomy; CPH: Cox proportional hazards; CSB: Chi-square binary event outcome; CSF: Chi-square binary fatal event outcome; CSO: Chi-square ordinal event outcome; HRT: hormone replacement therapy; MWU: Mann-Whitney U test; MT: median test; MLR: multiple linear regression; OLR: ordinal logistic regression; TT: t-test; Vascular: combination of stroke and MI; WR: win ratio test

### Sample size comparisons

The comparisons between sample sizes generated for the binary stroke outcome and the 4-level stroke/TIA outcome can be seen in Table 6. Sample size estimates were generated for ordinal logistic regression, [34] Mann-Whitney U test [35] and t-test [36], and compared with binary comparison of proportions [37]. In 13 comparator datasets and relative to binary sample size estimation, sample sizes were reduced by 34% for ordinal logistic regression and 82% for the Mann-Whitney-U test and t-test.

**Table 6:** Sample size comparisons (Stroke/TIA 4-level)

	Sample size				Multiplier			
Dataset	Binary	Ordinal	MWU	t-test	Binary	Ordinal	MWU	t-test
1	4528	4052	1194	1196	1	0.89	0.26	0.26
2	17,986	11,970	3326	3904	1	0.67	0.18	0.22
3	15,292	10,072	2744	2828	1	0.66	0.18	0.18
4	5436	2382	704	898	1	0.44	0.13	0.17
5	25,090	8666	2426	3362	1	0.35	0.097	0.13
6	30,450	10,164	2958	5070	1	0.33	0.097	0.17
13	13,510	12,610	3806	3754	1	0.93	0.28	0.28
18	23,090	22,110	6486	7194	1	0.96	0.28	0.31
30	15,934	1582	442	566	1	0.099	0.028	0.036
31	6934	1952	608	802	1	0.28	0.088	0.12
32	115,114	63,792	17,482	19,952	1	0.55	0.15	0.17
33	15,198	10,862	3388	3450	1	0.71	0.22	0.23
55	37,646	37,456	10,924	12,186	1	0.99	0.29	0.32
Median (Q1-Q3)	–	–	–	–	–	0.66 (0.35–0.89)	0.18 (0.097–0.26)	0.18 (0.17–0.26)

Abbreviations

MWU: Mann-Whitney U test; Q1: lower 25% quartile; Q3: upper 75% quartile

### Sensitivity analyses and statistical assumptions

The statistical assumptions for ordinal logistic regression (checked using the score test for proportional odds) were upheld (*p* > 0.05) in 51/56 (91.1%) datasets with 3-level stroke data and 30/35 (86%) datasets with the 4-level Stroke/TIA outcome. The sensitivity analysis assessing type I error, was performed on the 3-, 5- and 8-level stroke outcome, the 3-level MI outcome and the 4-level bleeding outcome. This analysis did not find any evidence of increased type I error rate for the Mann-Whitney U test or ordinal logistic regression (Supplementary Table 8).

## Discussion

We found that it is more efficient statistically to analyse vascular event data as several categories ordered by severity rather than as dichotomous event / no event data. The findings applied to both primary and secondary prevention trials, and to a variety of intervention types including blood pressure and lipid lowering, antithrombotics and carotid endarterectomy. Appropriate analysis approaches included the Mann-Whitney U test, ordinal logistic regression, bootstrapping, the Win ratio test and, for some ordinal scales, multiple linear regression (adjusted). In general, statistical regression models were more efficient if adjusted for prognostic factors than if performed unadjusted. Using ordinal outcome data and efficient analyses did not carry a risk of false positive findings. Finally, sample size estimations for ordered outcomes were significantly lower than for dichotomous events. These results extend our previous work based on published trial summary data [17].

In this study, there were two methods - the Mann-Whitney U test and win ratio test - which often appeared to be more efficient than the others. Although neither can be adjusted for covariates, extensions of these methods do allow for covariate adjustment [28–31]. Furthermore, though shown not to be as efficient as the ordinal methods, there are extensions that could be applied to the Cox proportional hazards model that enable adjustment for covariates in a manner that does not require the proportional hazards assumption for covariates [38].

The premise of this study is that an effective, or even hazardous, treatment alters both the risk and severity of an event. Although novel when considered across vascular prophylaxis, individual trials and meta-analyses have found this before, as seen in the Heart Protection Study with simvastatin, [39] and for HRT [40].

This study has a number of strengths. First, the study used individual participant data, not summary data, thereby allowing covariate adjusted analyses. Second, trials included both primary and secondary prevention studies and multiple intervention types thereby increasing the external validity of the findings. Third, ordered categorical outcomes have embedded dichotomies for poorer versus better outcome; for example, event versus no event, major event versus no major event and so on. Therefore, if statistical significance is shown, further closed testing methods can be applied to present results for the important dichotomies; this would be particularly useful for a trichotomous outcome for which the two embedded dichotomies are of primary interest.

### Limitations

However, there are also a number of limitations apparent. First, individual data were not shared for a majority of identified trials, a common problem in data pooling projects. Although non-availability of data might cause a systematic bias, the included trials involved both primary and secondary prevention studies, and a range of different interventions. Second, trials typically did not include sufficient information to allow 4 or more levels of ordinal data to be generated for MI and bleeding; some stroke trials allowed ordinal outcomes to be generated up to 9 levels. In principle, MI could be further categorised with the addition of unstable angina and angina, and MI could be divided into ST-elevation and non-ST elevation. Third, we did not use all of the statistical analysis methods that are relevant potentially for analysing ordered categorical data; rather we focussed on methods that are readily available in statistical text books and analysis software. Fourth, for the trials that had comparisons of two (or more) intervention groups to the same control group, the rankings of the *p*-values are not independent. However, this issue is unlikely to have had a significant impact on the final results because the rankings of methods were done within the respective comparisons. Fifth, the use of p-values (rather than quantifying effects) to compare the performance of methods and drawing conclusions is also a limitation as evidence-based decision-making is only partly influenced by p-values. However, this was necessary as different tests produce different quantifying effects and p-values offer a common currency. Furthermore, the ranking of p-values within comparisons is likely to be very similar to the ranking of standardized effect sizes. Last, there are other comparison methods that could be considered more suitable than the Freidman’s ANOVA and the Duncan’s test, such as a more generally applicable version of Friedman’s ANOVA test and a multivariate extension of the Wilcoxon signed ranks test [41], although each method has its own limitations. Nevertheless, these approaches were also utilised, for the same purpose, in the published optimising the analysis of acute stroke trials collaboration [19, 20].

### Future directions

Future work could include reviewing the statistical approaches utilised here using data from the TARDIS trial, which was the first prevention trial to use an ordinal event outcome as the primary [42]. In this trial data on other vascular events and their severity were also collected, therefore it would be possible to review these methods for these event outcomes. Another area to consider is in the case of where the occurrence of more than one type of event are of importance. In situations such as these, trials tend to use a binary composite outcome as the primary. An extension of the work undertaken here would therefore be to ascertain if it is suitable to include severity information in the composite outcome as well. Furthermore, there are certain statistical techniques that can analyse the individual effects of an intervention on multiple outcomes at once to determine a ‘global’ effect. It would therefore be of interest to determine if these methods would be more efficient than those that are typically used to analyse composite outcomes.

## Conclusions

In summary, vascular outcomes can be ordinal variables with ordering determined by the severity of the vascular event. This improves statistical efficiency as well as providing additional information for participants, public and healthcare practitioners. The approach appears to be relevant to all tested vascular interventions and outcomes (stroke, MI, major adverse cardiovascular events, bleeding). Further, where applicable, adjusted analyses add further statistical efficiency. The use of ordinal outcomes as primary outcomes could also lead to significant reductions in sample size. Future vascular prevention trials should consider whether to use ordered categorical outcomes and the statistical methods associated with those. If this approach is chosen the resulting trials could be smaller whilst retaining their original power, and would test the effect of interventions on severity, not just the absolute number of events. Implementation of this approach might then lead to smaller, shorter and less expensive vascular prophylaxis trials.

### What does this study add to the literature?

The use of ordinal vascular outcomes in prevention trials would improve statistical efficiency as well as providing additional information for participants, public and healthcare practitioners. If ordinal outcomes are used as primary outcomes this could lead to significant reductions in sample size. Furthermore, the resulting trials would not only be smaller but also retain their original power, and would test the effect of the intervention on severity, not just the number of events. Therefore, application of this approach could lead to smaller, shorter and less expensive vascular prophylaxis trials.

## Supplementary Information

**Additional file 1.**

## Data Availability

The data that support the findings of this study are not publicly available due to restrictions given in the data sharing contracts, and so can only be used under license for this current study only. Individual trial data are however available upon reasonable request to the Collaborators. Information regarding the appropriate collaborator to be contacted for each trial is listed in the ‘Trials, Collaborators & Acknowledgements’ section in the supplementary appendix.
