# Supplementary material for: Statistical reanalysis of vascular event outcomes in primary and secondary vascular prevention trials
Source: BMC Med Res Methodol. 2021 Oct 17;21:218. doi: 10.1186/s12874-021-01388-6 (PMC8520648; doi:10.1186/s12874-021-01388-6)
Supplement: Supplementary file 1 — Additional file 1. [file 12874_2021_1388_MOESM1_ESM.docx]

**Statistical reanalysis of vascular event outcomes in primary and secondary vascular prevention trials.**

**Supplementary Appendix**

The Optimising the Analysis of vascular Prevention trials Collaboration

**Writing committee**

Lisa J Woodhouse, Alan A Montgomery, Jonathan Mant, Barry R Davis, Ale Algra, Jean-Louis Mas, Jan A Staessen, Lutgarde Thijs, Andrew Tonkin, Adrienne Kirby, Stuart J Pocock, John Chalmers, Graeme J Hankey, J. David Spence, Peter Sandercock, Hans-Christoph Diener, Shinichiro Uchiyama, Nikola Sprigg, Philip M Bath

Correspondence: Professor Philip M Bath

Stroke Trials Unit

Mental Health & Clinical Neurosciences

School of Medicine

University of Nottingham

Queens Medical Centre

Nottingham, NG7 2UH

Tel: 0115 823 1765

Fax: 0115 823 1767

Email: [philip.bath@nottingham.ac.uk](mailto:philip.bath@nottingham.ac.uk)

Table count: 8

Figure count: 1

**Trials, Collaborators & Acknowledgements**

4S: TR Pedersen & **MERCK** (Kenilworth, USA)

ACAS: JF Toole & **NINDS** (Bethesda, USA)

ACCORD: R Byington & **NHLBI** (Bethesda, USA)

AFCAPS/TEXCAPS: AM Gotto Jr & **MERCK** (Kenilworth, USA)

ALLHAT: BR Davis & **NHLBI** (Bethesda, USA)

APOLLO: AGG Turpie & **GlaxoSmithKline** (Brentford, UK)

BAFTA: **J Mant** & K Fletcher

CARE: **F Sacks, BR Davis** & L Moye

Dutch-TIA: **A Algra**

EAFT: **PJ Koudstaal**

ENOS: **PM Bath** & N Sprigg

ESPRIT: **A Algra**

ESPS 1: A Lowenthal, **H-C Diener**

ESPS 2: **H-C Diener**

EVA-3S: **J-L Mas** & D Calvet

EWPHE: **JA Staessen** & L Thijs

IST-1: P Sandercock (**Data available online**)

JASAP: S Uchiyama & **Boehringer Ingelheim** (Ingelheim am Rhein, Germany)

LIPID: **A Tonkin** & A Kirby

MRC 1: **SJ Pocock**

MRC 2: **SJ Pocock**

NASCET: H Barnett, GG Ferguson & **NINDS** (Bethesda, USA)

OASIS-6: S Yusuf & **GlaxoSmithKline** (Brentford, UK)

PETRO: MD Ezekowitz & **Boehringer Ingelheim** (Ingelheim am Rhein, Germany)

RE-LY: SJ Connolly, MD Ezekowitz, S Yusuf, L Wallentin & **Boehringer Ingelheim** (Ingelheim am Rhein, Germany)

PROGRESS: **J Chalmers** & H Arima

PROFESS: H-C Diener, R Sacco, S Yusuf & **Boehringer Ingelheim** (Ingelheim am Rhein, Germany)

SHEP: BR Davis & **NHLBI** (Bethesda, USA)

SPIRIT: **A Algra**

SYST-EUR: **JA Staessen** & L Thijs

TARDIS: **PM Bath** & N Sprigg

VISP: JF Toole, JD Spence & **NINDS** (Bethesda, USA)

VITATOPS: **GJ Hankey**, Q Yi & JW Eikelboom

WHI project (WHI 1 & 2): **S Wassertheil-Smoller**

**NB:** Data were shared after submission of formal application or request to the collaborators highlighted in bold.

**Links for data repositories**

Boehringer Ingelheim and GlaxoSmithKline: [www.clinicalstudydatarequest.com](http://www.clinicalstudydatarequest.com)

IST-1: <https://trialsjournal.biomedcentral.com/articles/10.1186/1745-6215-12-101>

MERCK: <http://engagezone.msd.com/ds_documentation.php>

NHLBI: <https://biolincc.nhlbi.nih.gov/home/>

NINDS: <https://www.ninds.nih.gov/Current-Research/Research-Funded-NINDS/Clinical-Research/Archived-Clinical-Research-Datasets>

**Search strategy**

Eligible vascular prevention trials were sought, during the period May 2013 to December 2016, using the following electronic search engines:

- Cochrane Library
- PubMed and
- Google Scholar

(search terms are shown in Supplementary Table 1)

**List of Tables and Figures**

[Table 1: Search terms for Cochrane Library, PubMed and Google Scholar 5](#_Toc70075963)

[Table 2: List of included trials by indication and whether primary or secondary preventions 6](#_Toc70075964)

[Table 3: Information on included trials 7](#_Toc70075965)

[Table 4. Ordered categorical severity levels for stroke, myocardial infarction, major adverse cardiovascular events and bleeding, and the number of datasets for each outcome 9](#_Toc70075966)

[Table 5. Example analysis process – based on 4-level outcome (fatal stroke/ non-fatal stroke/ TIA/ no event) 10](#_Toc70075967)

[Table 6: Duncan’s test analysis of p-value ranks for 3-level 11](#_Toc70075968)

[Table 7: Duncan’s test analysis of p-value ranks for 3-level bleeding 12](#_Toc70075969)

[Table 8. Type 1 error analyses 13](#_Toc70075970)

[Figure 1a: Box plot showing distribution of p-value ranks by test - stroke/TIA 4-level 14](#_Toc70075971)

[Figure 1b: Box plot showing distribution of p-values by test - stroke/TIA 4-level 14](#_Toc70075972)

[Figure 2: Box plot showing distribution of ranks by subgroups for 4-level stroke/TIA 15](#_Toc70075973)

# Table 1: Search terms for Cochrane Library, PubMed and Google Scholar

| **Search terms** | **Combinations** |
| --- | --- |
| 1. “Prevention” |  |
| 1. “Primary prevention” |  |
| 1. “Secondary prevention” |  |
| 1. “Vascular” | & (1) or (2) or (3) |
| 1. “Cardiovascular” | & (1) or (2) or (3) |
| 1. “Cerebrovascular” | & (1) or (2) or (3) |
| 1. “Stroke” | & (1) or (2) or (3) |
| 1. “TIA” | & (1) or (2) or (3) |
| 1. “Myocardial Infarction” | & (1) or (2) or (3) |
| 1. “Angina” | & (3) & (16) or (19) or (24) |
| 1. “Bleeding” | & (16) or (22) |
| 1. “Venous thromboembolism” | & (1) & (22) |
| 1. “Carotid endarterectomy” | & (1) |
| 1. “Carotid stenting” | & (1) |
| 1. “Carotid stenosis” | & (13) or (14) |
| 1. “Antiplatelets” | & (1) or (2) or (3) & (6) or (7) |
| 1. “Aspirin” | & (6) or (7) or (8) |
| 1. “Clopidogrel” | & (6) or (7) or (8) |
| 1. “Statins” | & (1) & (5) or (6) or (7) or (9) |
| 1. “Lipid lowering” | & (2) & (5) or (6) or (7) or (9) |
| 1. “Hyperlipidaemia” | & (2) & (5) or (6) or (7) or (9) |
| 1. “Anticoagulants” | & (1) or (2) or (3) & (5) or (6) or (7) or (9) |
| 1. “Atrial fibrillation” | & (1) or (2) & (22) |
| 1. “Antihypertensives” | & (1) or (2) or (3) & (5) or (6) or (7) or (9) |
| 1. “Hypertension” | & (2) & (5) or (6) or (7) or (9) & (24) |
| 1. “Glucose lowering” | & (1) & (5) or (6) or (7) or (9) & (27) |
| 1. “Diabetes” | & (2) & (5) or (6) or (7) or (9) & (26) |
| 1. “B Vitamins” | & (5) or (6) or (7) |
| 1. “Hormone replacement therapy” | & (5) or (6) or (7) or (9) |

# Table 2: List of included trials by indication and whether primary or secondary preventions

| **Intervention** | **Type of trial** | |
| --- | --- | --- |
|  | **Primary** | **Secondary** |
| Antiplatelets |  | Dutch-TIA^†^[1]  ESPRIT^†^[2]  ESPS 1[3]  ESPS 2[4]  JASAP[5]  PROFESS[6]  TARDIS[7] |
| Anticoagulants | BAFTA[8]  APOLLO[9]  PETRO[10]  RE-LY[11] | EAFT[12]  OASIS-6[13]  SPIRIT[14]  IST-1*[15] |
| Antihypertensives | ALLHAT[16]  EWPHE[17]  INDANA MRC 1[18]  INDANA MRC 2[19]  SHEP[20]  SYST-EUR[21] | PROGRESS[22]  ENOS*[23] |
| Carotid Endarterectomy | ACAS[24] | EVA-3S[25]  NASCET[26] |
| Vitamins |  | VISP[27]  VITATOPS[28] |
| HRT^1^ | WHI 1[29]  WHI 2[30] |  |
| Statins | AFCAPS/TEXCAPS[31] | 4S[32]  LIPID[33]  CARE[34] |
| Glucose lowering | ACCORD[35] |  |

^1^HRT: Hormone replacement therapy.

*Acute trials that collected data on vascular events.

^†^Participants in these trials could also be randomised to receive either an antihypertensive (DUTCH-TIA) or an anticoagulant (ESPRIT). Trials have been listed according to their primary intervention.

# Table 3: Information on included trials

| Trial | Year | Class | Treatment | Control | Size | Length of follow-up | Data source |
| --- | --- | --- | --- | --- | --- | --- | --- |
| 4S[32] | 1994 | Statins | Simvastatin | Placebo | 4444 | 5.4 years | Merck |
| ACAS[24] | 1995 | CEA | CEA | No surgery | 1662 | 2.7 years | NINDS |
| ACCORD[35] | 2008 | GL | Intensive GL | Standard | 10251 | 3.5 years | NHLBI |
| AFCAPS/TEXCAPS[31] | 1998 | Statins | Lovastatin | Placebo | 6605 | 5.2 years | Merck |
| ALLHAT[16] | 2002 | AHT | CCB or ACE-I | Diuretic | 33357 | 4.9 years | NHLBI |
| APOLLO[9] | 2007 | ACT | FS | Placebo | 1309 | 1 month | CTDR |
| BAFTA[8] | 2007 | ACT | Warfarin | A | 973 | 2.7 years | CI |
| CARE[34] | 1996 | Statins | Pravastatin | Placebo | 4159 | 5 years | CI |
| DUTCH-TIA[1] | 1991 | APT | A (283mg per day)/ BB | A (30mg per day)/ Placebo | 3131 | 2.6 years | CI |
| EAFT[12] | 1993 | ACT | ACT or Aspirin | Placebo | 1007 | 2.3 years | CI |
| ENOS[23] | 2014 | AHT | GTN | No GTN | 4011 | 3 months | CI |
| ESPRIT[2] | 2007 | APT | AD/ACT | A | 1068 | 4.6 years | CI |
| ESPS 1[3] | 1990 | APT | AD | Placebo | 2500 | 2 years | CI |
| ESPS 2[4] | 1996 | APT | A, D or AD | Placebo | 6602 | 2 years | CI |
| EVA-3S[25] | 2006 | CEA | CEA | Stenting | 527 | 4 years | CI |
| EWPHE[17] | 1985 | AHT | H + T | Placebo | 840 | 4.7 years | CI |
| INDANA MRC1[18] | 1985 | AHT | Bendro or Prop | Placebo | 17354 | 5 years | CI |
| INDANA MRC2[19] | 1992 | AHT | Diuretic or BB | Placebo | 4396 | 5.8 years | CI |
| IST-1[15] | 1997 | ACT | Heparin | No heparin | 19435 | 6 months | Online |
| JASAP[5] | 2011 | APT | Aggrenox | A | 1294 | 4 months | CTDR |
| LIPID[33] | 1998 | Statins | Pravastatin | Placebo | 9014 | 6.1 years | CI |
| NASCET[26] | 1998 | CEA | CEA | No surgery | 2267 | 5 years | NINDS |
| OASIS 6[13] | 2006 | ACT | FS | Placebo/Heparin | 12092 | 6 months | CTDR |
| PETRO[10] | 2007 | ACT | Dabigatran | Warfarin | 502 | 3 months | CTDR |
| PROFESS[6] | 2008 | APT | AD | C | 20332 | 2.5 years | CTDR |
| PROGRESS[22] | 2001 | AHT | Perindopril | Placebo | 6105 | 4 years | CI |
| RE-LY[11] | 2009 | ACT | Dabigatran | Warfarin | 18113 | 2 years | CTDR |
| SHEP[20] | 2000 | AHT | Chlorthalidone | Placebo | 4736 | 4.5 years | NHLBI |
| SPIRIT[14] | 1997 | ACT | ACT | A | 1316 | 14 months | CI |
| SYST-EUR[21] | 1997 | AHT | Nitrendipine | Placebo | 6403 | 2 years | CI |
| TARDIS[7] | 2017 | APT | ACD | AD or C | 3996 | 3 months | CI |
| VISP[27] | 2004 | Vitamins | High FA, B_6_ + B_12_ | Low FA, B_6_ + B_12_ | 3680 | 2 years | NINDS |
| VITATOPS[28] | 2010 | Vitamins | B vitamins | Placebo | 8164 | 3.4 years | CI |
| WHI 1[29] | 2002 | HRT | Estrogen + Progestin | Placebo | 16608 | 5.2 years | CI |
| WHI 2[30] | 2004 | HRT | Estrogen | Placebo | 10739 | 5.2 years | CI |

Abbreviations

ACT: Anticoagulants, AHT: Antihypertensives, APT: Antiplatelets, CEA: Carotid Endarterectomy, GL: Glucose lowering, HRT: Hormone replacement therapy, CCB: Calcium channel blocker, ACE-I: Angiotensin-converting enzyme inhibitor, FS: Fondaparinux Sodium, A: Aspirin, D: Dipyridamole, C: Clopidogrel, H + T: Hydrochlorothiazide plus Triamterene, Bendro: Bendroflumethiazide, Prop: Propranolol, BB: Beta blocker, FA: Folic acid, CI: Chief Investigator, CTDR: clinical trials data repository, NHLBI: National Heart, Lung and Blood Institute, NINDS: National Institute of Neurological Disorders and Stroke.

# Table 4. Ordered categorical severity levels for stroke, myocardial infarction, major adverse cardiovascular events and bleeding, and the number of datasets for each outcome

| Levels | Stroke | MI | MACE | Bleeding |
| --- | --- | --- | --- | --- |
| 3 | Fatal / non-fatal / none (n=56) | Fatal / non-fatal / none (n=47) | Fatal / non-fatal / none (n=47) | Fatal / non-fatal / none (n=32) |
| 4 | Fatal / severe / moderate-mild / none (n=23) |  |  | Fatal / severe / moderate-mild / none (n=26) |
| 4 | Fatal / non-fatal / TIA / none (n=35) |  |  |  |
| 5 | Fatal / severe / moderate / mild / none (n=16) |  |  | Fatal / severe / moderate / minor / none (n=13) |
| 5 | Fatal / severe / moderate-mild / TIA / none (n=17) |  |  |  |
| 6 | Fatal / severe / moderate / mild / TIA / none (n=13) |  |  |  |
| 8 | Fatal / mRS=5 / mRS=4 / mRS=3 / mRS=2 / mRS=1 / mRS=0 / none (n=12) |  |  |  |
| 9 | Fatal / mRS=5 / mRS=4 / mRS=3 / mRS=2 / mRS=1 / mRS=0 / TIA / none (n=12) |  |  |  |

Abbreviations

MI: Myocardial infarction; MACE: Major cardiovascular events; mRS: modified Rankin Scale

Table 5. Example analysis process – based on 4-level outcome (fatal stroke/ non-fatal stroke/ TIA/ no event)

| 1. For each of the 35 comparator datasets with available data regarding fatal stroke, non-fatal stroke and TIA the following outcomes were created:    1. **Binary (event/no event)** – analysed using the following approaches: Unadjusted – Chi-Square test, Cox Proportional hazards; Adjusted – binary logistic regression and Cox proportional hazards    2. **Binary (fatal event/ no fatal event)** – analysed using Chi-Square test and used for the Win ratio test    3. **Binary (non-fatal event/ no non-fatal event)** – for use in the Win ratio test only    4. **Binary (TIA/ no TIA)** – for use in Win ratio test only    5. **Ordinal 4-level**– analysed using the following approaches: Unadjusted – ordinal logistic regression, Mann-Whitney U test, Cochran-Armitage trend test, Median test, Chi-Square test, t-test, and bootstrapping of the mean rank; Adjusted – ordinal logistic regression and multiple linear regression. | | | | | | | | | | | | | | | |
| --- | --- | --- | --- | --- | --- | --- | --- | --- | --- | --- | --- | --- | --- | --- | --- |
| 1. For each dataset:    1. All of the tests are performed on the corresponding outcome and the p-values are extracted to a new dataset, e.g. | | | | | | | | | | | | | | | |
| **Dataset** | **Chi-Square (Fatal)** | **Chi-Square (Binary)** | **Adjusted BLR** | **Adjusted CPH** | **Adjusted OLR** | **Adjusted MLR** | **Un-adjusted CPH** | **C-A trend test** | **Chi-Square (Ordinal)** | **Un-adjusted OLR** | **t-test** | **Mann-Whitney U** | **Median test** | **Boot-strapping** | **Win ratio** |
| X | 0.91 | 0.0074 | 0.0052 | 0.0039 | 0.0011 | 0.0027 | 0.0049 | 0.0031 | 0.43 | 0.0014 | 0.0030 | 0.0013 | 0.0017 | 0.0037 | 0.024 |
| - 1. Then each of the p-values is ranked 1 to 15, from smallest to largest, e.g. | | | | | | | | | | | | | | | |
| **Dataset** | **Chi-Square (Fatal)** | **Chi-Square (Binary)** | **Adjusted BLR** | **Adjusted CPH** | **Adjusted OLR** | **Adjusted MLR** | **Un-adjusted CPH** | **C-A trend test** | **Chi-Square (Ordinal)** | **Un-adjusted OLR** | **t-test** | **Mann-Whitney U** | **Median test** | **Boot-strapping** | **Win ratio** |
| X | 15 | 12 | 11 | 9 | 1 | 5 | 10 | 7 | 14 | 3 | 6 | 2 | 4 | 8 | 13 |
| 1. This process is completed for all 35 comparator datasets and these are then aggregated into a single dataset, e.g. | | | | | | | | | | | | | | | |
| **Dataset** | **Chi-Square (Fatal)** | **Chi-Square (Binary)** | **Adjusted BLR** | **Adjusted CPH** | **Adjusted OLR** | **Adjusted MLR** | **Un-adjusted CPH** | **C-A trend test** | **Chi-Square (Ordinal)** | **Un-adjusted OLR** | **t-test** | **Mann-Whitney U** | **Median test** | **Boot-strapping** | **Win ratio** |
| X | 15 | 12 | 11 | 9 | 1 | 5 | 10 | 7 | 14 | 3 | 6 | 2 | 4 | 8 | 13 |
| Y | 15 | 13 | 10 | 11 | 3 | 6 | 12 | 5 | 14 | 2 | 7 | 1 | 8 | 9 | 4 |
| $\vdots$ | $\vdots$ | $\vdots$ | $\vdots$ | $\vdots$ | $\vdots$ | $\vdots$ | $\vdots$ | $\vdots$ | $\vdots$ | $\vdots$ | $\vdots$ | $\vdots$ | $\vdots$ | $\vdots$ | $\vdots$ |
| 1. This new dataset is then analysed using Friedman’s ANOVA and the Duncan’s test to determine if there is a difference in the average ranking of each test and the ordering/grouping of tests respectively. | | | | | | | | | | | | | | | |
| 1. This process is repeated for each ordinal outcome in Supplementary Table 4. | | | | | | | | | | | | | | | |

Table 6: Duncan’s test analysis of p-value ranks for 3-level myocardial infarction based on 47 comparator datasets (p=0.010)

| **Test** |  | | | | | **Mean rank** |
| --- | --- | --- | --- | --- | --- | --- |
| MWU (3-level) | A |  |  |  |  | 6.04 |
| Bootstrapping (3-level) | A | B |  |  |  | 6.47 |
| CA trend (3-level) | A | B | C |  |  | 6.62 |
| t-test (3-level) | A | B | C |  |  | 6.91 |
| OLR (3-level) | A | B | C |  |  | 6.94 |
| Adj. OLR (3-level) | A | B | C |  |  | 7.09 |
| Adj. MLR (3-level) | A | B | C |  |  | 7.09 |
| Adj. BLR (Binary) | A | B | C |  |  | 7.55 |
| Chi-Square (3-level) | A | B | C |  |  | 8.02 |
| CPH (Binary) |  | B | C |  |  | 8.21 |
| Adj. CPH (Binary) |  |  | C | D |  | 8.53 |
| Win ratio* |  |  | C | D |  | 8.55 |
| Chi-square (Binary) |  |  |  | D | E | 10.17 |
| Median test (3-level) |  |  |  |  | E | 10.89 |
| Chi-square (Binary fatal) |  |  |  |  | E | 10.91 |

Abbreviations

MWU: Mann-Whitney U test, OLR: ordinal logistic regression: CA trend: Cochran-Armitage trend test, MLR: multiple linear regression, BLR: binary logistic regression, CPH: Cox proportional hazards

* Combined binary outcomes including (from most to least clinically important): fatal MI (Yes/No) and Non-fatal MI (Yes/No)

Table 7: Duncan’s test analysis of p-value ranks for 3-level bleeding based on 32 comparator datasets (p<0.0001)

| **Test** |  | | | **Mean rank** | |
| --- | --- | --- | --- | --- | --- |
| Adj. MLR (3-level) | A |  |  | 5.38 |  |
| CA trend (3-level) | A | B |  | 5.91 |  |
| Win Ratio* | A | B |  | 6.28 |  |
| MWU (3-level) | A | B |  | 6.72 |  |
| t-test (3-level) | A | B |  | 6.75 |  |
| Adj. CPH (Binary) | A | B |  | 6.84 |  |
| Bootstrapping (3-level) | A | B |  | 7.00 |  |
| Adj. BLR (Binary) | A | B |  | 7.09 |  |
| Adj. OLR (3-level) | A | B |  | 7.13 |  |
| CPH (Binary) | A | B |  | 7.56 |  |
| OLR (3-level) |  | B |  | 7.97 |  |
| Chi-square (Binary fatal) |  |  | C | 10.97 |  |
| Chi-square (Binary) |  |  | C | 11.03 |  |
| Chi-square (3-level) |  |  | C | 11.31 |  |
| Median test (3-level) |  |  | C | 12.06 |  |

Abbreviations

MWU: Mann-Whitney U test, OLR: ordinal logistic regression: CA trend: Cochran-Armitage trend test, MLR: multiple linear regression, BLR: binary logistic regression, CPH: Cox proportional hazards

* Combined binary outcomes including (from most to least clinically important): fatal bleeding event (Yes/No) and Non-fatal bleeding event (Yes/No)

# Table 8. Type 1 error analyses

|  |  |  | MWU | | OLR | |
| --- | --- | --- | --- | --- | --- | --- |
| Outcome | Level | Comparator dataset | N samples | P | N samples | P |
| Stroke | 3 | ALLHAT[16] | 57/1000 | 0.15 | 57/1000 | 0.15 |
|  | 3 | MRC-1[18] | 52/1000 | 0.61 | 50/1000 | 1.00 |
|  | 5 | ACAS[24] | 48/1000 | 1.00 | 47/1000 | 1.00 |
|  | 5 | EAFT[12] | 53/1000 | 0.33 | 51/1000 | 0.44 |
|  | 8 | ESPS-2[4] | 50/1000 | 1.00 | 50/1000 | 1.00 |
|  | 8 | NASCET[26] | 50/1000 | 1.00 | 50/1000 | 1.00 |
| MI | 3 | LIPID[33] | 54/1000 | 0.28 | 54/1000 | 0.28 |
|  | 3 | SHEP[20] | 37/1000 | 1.00 | 37/1000 | 1.00 |
| Bleeding | 4 | SPIRIT[14] | 49/1000 | 1.00 | 49/1000 | 1.00 |
|  | 4 | TARDIS[7] | 46/1000 | 1.00 | 46/1000 | 1.00 |

Abbreviations

MWU: Mann-Whitney U test; OLR: ordinal logistic regression

#
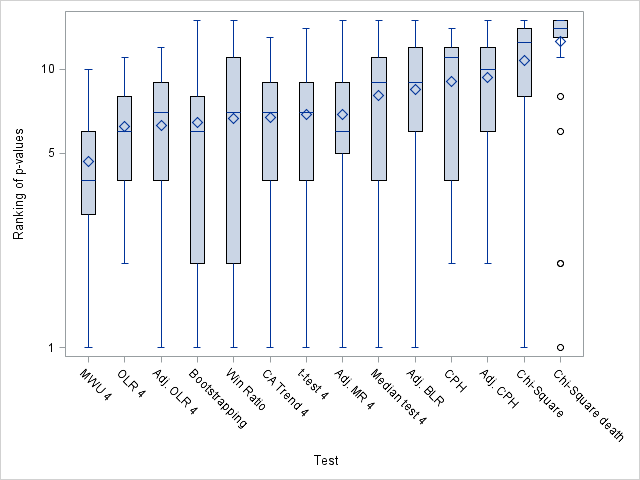
Figure 1a: Box plot showing distribution of p-value ranks by test - stroke/TIA 4-level

# Figure 1b: Box plot showing distribution of p-values by test - stroke/TIA 4-level


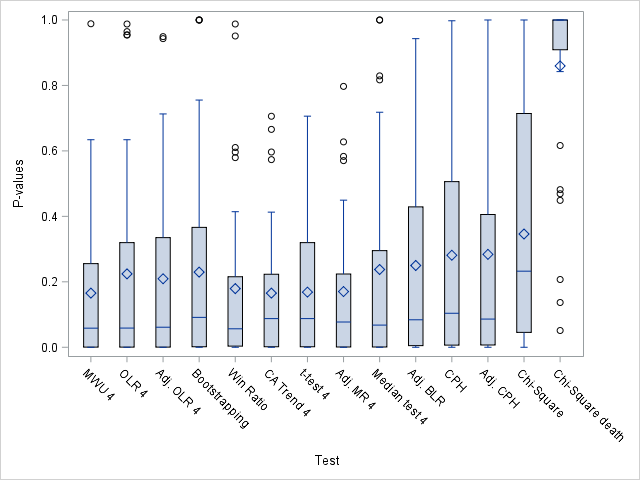


# Figure 2: Box plot showing distribution of ranks by subgroups for 4-level stroke/TIA


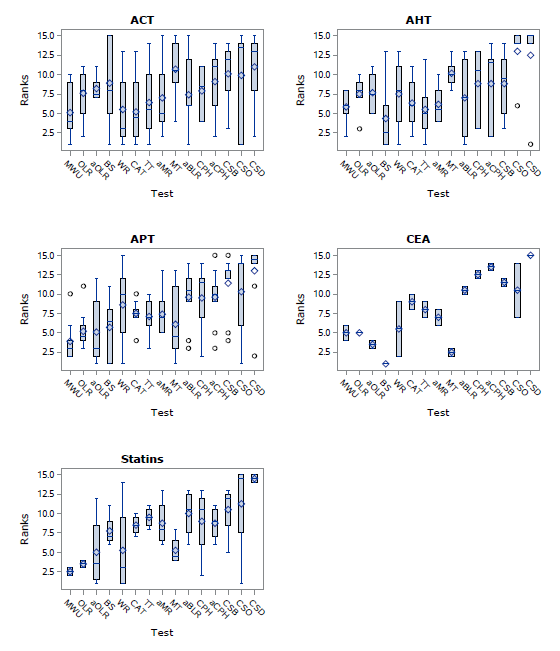


Abbreviations

MWU: Mann-Whitney U test; OLR: ordinal logistic regression; aOLR: adjusted ordinal logistic regression; BS: Bootstrapping; WR: Win ratio test; CAT: Cochran-Armitage trend test; TT: t-test; aMR: adjusted multiple linear regression; MT: Median test; aBLR: adjusted binary logistic regression; CPH: Cox proportional hazards model; aCPH: adjusted Cox proportional hazards model; CSB: Binary Chi-Square test; CSO: Ordinal Chi-Square test; CSD: Binary Chi-Square test performed on fatal/no-fatal event outcome.

**References**

1. Algra, A., et al., *Trial of secondary prevention with atenolol after transient ischemic attack or nondisabling ischemic stroke. The Dutch TIA Trial Study Group.* Stroke, 1993. **24**(4): p. 543-548.

2. Algra, A., *Medium intensity oral anticoagulants versus aspirin after cerebral ischaemia of arterial origin (ESPRIT): a randomised controlled trial.* Lancet Neurology, 2007. **6**(2): p. 115-124.

3. ESPS Group, *European Stroke Prevention Study.* Stroke, 1990. **21**: p. 1122-1130.

4. Diener, H.C., et al., *European Stroke Prevention Study. 2. Dipyridamole and acetylsalicylic acid in the secondary prevention of stroke.* J Neurol Sci, 1996. **143**(1-2): p. 1-13.

5. Uchiyama, S., et al., *The Japanese aggrenox (extended-release dipyridamole plus aspirin) stroke prevention versus aspirin programme (JASAP) study: a randomized, double-blind, controlled trial.* Cerebrovascular Diseases, 2011. **31**(6): p. 601-13.

6. Sacco, R.L., et al., *Aspirin and extended-release dipyridamole versus clopidogrel for recurrent stroke.* New England Journal of Medicine, 2008. **359**(12): p. 1238-1251.

7. Bath, P.M., et al., *Antiplatelet therapy with aspirin, clopidogrel, and dipyridamole versus clopidogrel alone or aspirin and dipyridamole in patients with acute cerebral ischaemia (TARDIS): a randomised, open-label, phase 3 superiority trial.* Lancet, 2018. **391**(10123): p. 850-859.

8. Mant, J., et al., *Warfarin versus aspirin for stroke prevention in an elderly community population with atrial fibrillation (the Birmingham Atrial Fibrillation Treatment of the Aged Study, BAFTA): a randomised controlled trial.* Lancet, 2007. **370**(9586): p. 493-503.

9. Turpie, A.G., et al., *Fondaparinux combined with intermittent pneumatic compression vs. intermittent pneumatic compression alone for prevention of venous thromboembolism after abdominal surgery: a randomized, double-blind comparison.* J Thromb Haemost, 2007. **5**(9): p. 1854-61.

10. Ezekowitz, M.D., et al., *Dabigatran with or without concomitant aspirin compared with warfarin alone in patients with nonvalvular atrial fibrillation (PETRO Study).* Am J Cardiol, 2007. **100**(9): p. 1419-26.

11. Connolly, S.J., et al., *Dabigatran versus Warfarin in Patients with Atrial Fibrillation.* New England Journal of Medicine, 2009. **361**(12): p. 1139-1151.

12. EAFT (European Atrial Fibrillation Trial) Study Group, *Secondary prevention in non-rheumatic atrial fibrillation after TIA or minor stroke.* Lancet, 1993. **342**(8882): p. 1255-1262.

13. Yusuf, S., et al., *Effects of fondaparinux on mortality and reinfarction in patients with acute ST-segment elevation myocardial infarction: the OASIS-6 randomized trial.* JAMA, 2006. **295**(13): p. 1519-30.

14. Algra, *A randomized trial of anticoagulants versus aspirin after cerebral ischemia of presumed arterial origin. The Stroke Prevention in Reversible Ischemia Trial (SPIRIT) Study Group.* Annals of Neurology, 1997. **42**(6): p. 857-865.

15. Sandercock, P., et al., *The International Stroke Trial (IST): A randomised trial of aspirin, subcutaneous heparin, both, or neither among 19 435 patients with acute ischaemic stroke.* Lancet, 1997. **349**(9065): p. 1569-1581.

16. Trial, A.O.a.C.f.t.A.C.R.G.T.A.a.L.-L.T.t.P.H.A., *Major outcomes in high-risk hypertensive patients randomized to angiotensin-converting enzyme inhibitor or calcium channel blocker vs diuretic: The Antihypertensive and Lipid-Lowering Treatment to Prevent Heart Attack Trial (ALLHAT).* JAMA, 2002. **288**(23): p. 2981-97.

17. Bulpitt, C.J. and J.A. Staessen, *Mortality and morbidity results from the european Working Party on High Blood Pressure in the Elderly Trial (EWPHE).* Drugs, 1986. **31**(SUPPL. 1): p. 29-39.

18. Medical Research Council Working Party, *MRC trial of treatment of mild hypertension: principal results. Medical Research Council Working Party.* British Medical Journal Clin Res Ed, 1985. **291**(6488): p. 97-104.

19. MRC Working Party, *Medical Research Council trial of treatment of hypertension in older adults: principal results.* British Medical Journal, 1992. **304**: p. 405-412.

20. SHEP Cooperative Research Group, *Prevention of stroke by antihypertensive drug treatment in older persons with isolated systolic hypertension. Final results of the Systolic Hypertension in the Elderly Program (SHEP).* Jama, 1991. **265**(24): p. 3255-64.

21. Staessen, J.A., et al., *Randomised double-blind comparison of placebo and active treatment for older patients with isolated systolic hypertension.* Lancet, 1997. **350**: p. 757-764.

22. PROGRESS Collaborative Group, *Randomised trial of a perindopril-based blood-pressure-lowering regimen among 6105 individuals with previous stroke or transient ischaemic attack.* Lancet, 2001. **358**: p. 1033-1041.

23. Bath, P.M., et al., *Efficacy of nitric oxide, with or without continuing antihypertensive treatment, for management of high blood pressure in acute stroke (ENOS): a partial-factorial randomised controlled trial.* Lancet, 2015. **385**(9968): p. 617-28.

24. *Endarterectomy for asymptomatic carotid artery stenosis. Executive Committee for the Asymptomatic Carotid Atherosclerosis Study.* JAMA, 1995. **273**(18): p. 1421-8.

25. Mas, J.-L., et al., *Endarterectomy versus stenting in patients with symptomatic severe carotid stenosis.* The New England Journal of Medicine, 2006. **355**: p. 1660-71.

26. North American Symptomatic Carotid Endarterectomy Trial Collaborators, *Beneficial effect of carotid endarterectomy in symptomatic patients with high-grade carotid stenosis.* New England Journal of Medicine, 1991. **325**: p. 445-453.

27. Toole, J.F., et al., *Lowering homocysteine in patients with ischemic stroke to prevent recurrent stroke, myocardial infarction, and death.* JAMA, 2004. **291**(5): p. 565-575.

28. Group., V.T.S., *B vitamins in patients with recent transient ischaemic attack or stroke in the VITAmins TO Prevent Stroke (VITATOPS) trial: a randomised, double-blind, parallel, placebo-controlled trial.* Lancet Neurol, 2010. **9**(9): p. 855-65.

29. Writing group for the women's health initiative investigators, *Risk and benefits of estrogen plus progestin in healthy menopausal women. Principal results from the women's health initiatve randomized controlled trial.* JAMA, 2002. **288**(3): p. 321-333.

30. The Women's Health Initiative Steering, C., *Effects of conjugated equine estrogen in postmenopausal women with hysterectomy: The women's health initiative randomized controlled trial.* JAMA, 2004. **291**(14): p. 1701-1712.

31. Downs, J.R., et al., *Primary prevention of acute coronary events with lovastatin in men and women with average cholesterol levels: results of AFCAPS/TexCAPS. Air Force/Texas Coronary Atherosclerosis Prevention Study.* JAMA, 1998. **279**(20): p. 1615-22.

32. Scandinavian Simvastatin Survival Study Group, *Randomised trial of cholesterol lowering in 4444 patients with coronary heart disease: the Scandinavian Simvastatin Survival Study (4S).* Lancet, 1994. **344**: p. 1383-1389.

33. Tonkin, A.M., et al., *Effects of pravastatin in 3260 patients with unstable angina: Results from the LIPID study.* Lancet, 2000. **356**(9245): p. 1871-1875.

34. Sacks, F.M., et al., *The effect of pravastatin on coronary events after myocardial infarction in patients with average cholesterol levels.* N.Engl.J.Med., 1996. **335**: p. 1001-1009.

35. Gerstein, H.C., et al., *Effects of intensive glucose lowering in type 2 diabetes.* N Engl J Med, 2008. **358**(24): p. 2545-59.
